# The Association between* C9orf72* Repeats and Risk of Alzheimer's Disease and Amyotrophic Lateral Sclerosis: A Meta-Analysis

**DOI:** 10.1155/2016/5731734

**Published:** 2016-06-08

**Authors:** Li Shu, Qiying Sun, Yuan Zhang, Qian Xu, Jifeng Guo, Xinxiang Yan, Beisha Tang

**Affiliations:** Xiangya Hospital, Central South University, Hunan, China

## Abstract

*C9orf72* is the most common genetic cause of amyotrophic lateral sclerosis (ALS) and frontotemporal dementia (FTD) in Caucasian populations. However, the relationship between* C9orf72* repeats and Alzheimer's disease (AD) was not clear. Additionally, there were few articles assessing* C9orf72 *in other ethnicities with ALS. In this meta-analysis, we aimed to investigate the relationship between* C9orf72* repeat expansions (≥30 repeats) and intermediate repeat copies (20–29 repeats) and AD or ALS. The results suggested positive correlations between* C9orf72* repeat expansions and the risk of Alzheimer's disease (OR = 6.36, 95% CI = 3.13–12.92, and *p* < 0.00001), while intermediate repeat copies of* C9orf72* gene were not associated with the risk of the disease.* C9orf72* repeat expansions were positively correlated with the risk of familial and sporadic ALS (OR = 293.25, 95% CI = 148.17–580.38, and *p* < 0.00001; OR = 35.57, 95% CI = 19.61–64.51, and *p* < 0.00001). There was a positive correlation between the gene variations and ALS risk among Caucasians and Asians (OR = 57.56, 95% CI = 36.73–90.22, and *p* < 0.00001; OR = 6.35, 95% CI = 1.39–29.02, and *p* = 0.02).

## 1. Introduction

In 2006, two researchers reported a locus on the short arm of chromosome 9 which could be a causative for FTD, ALS, and ALS/FTD [1]. In 2011, hexanucleotide (GGGGCC) repeat expansions in the noncoding region of* C9orf72* were confirmed to be the most common mutation of FTD, ALS, and ALS/FTD [2]. The frequencies of the mutation account for 29%, 50%, and 88% of the patients, respectively [3].

In human genome,* C9orf72 *gene is located on chromosome 9p21, spanning 27,546,543–27,573,864 base pairs. It encodes 11 exons and GGGGCC (G4C2) exists between noncoding exons 1a and 1b [4]. Some researchers suggested that G4C2 ≥ 30 repeats were pathological repeat expansions, while <20 repeat units were normal [5]. In neurodegenerative diseases such as ALS-FTD, the G4C2 repeat copies of* C9orf72* could reach 700–1600 units [4]. In addition, some researchers demonstrated that G4C2 intermediate copies (20–29 repeats) could also contribute to the risk of neurodegenerative diseases [6]. The disease mechanisms of how* C9orf72* expanded repeats lead to these diseases are still unknown. The possible hypotheses may be the loss of function of C9orf72 protein, the accumulation of toxic RNA foci, and the Repeat Associated Non-ATG Initiated Translation (RAN-Translation) [7–10].

Substantial clinical and pathological characteristics overlap among the common neurodegenerative diseases, FTD (frontotemporal dementia), ALS (amyotrophic lateral sclerosis), and AD (Alzheimer's disease). For example, ALS patients with* C9orf72 *repeat expansions can present with dementia which were common in FTD and AD patients [11, 12]. Tau positive pathology which is typical for AD can be found in FTD patients [13]. Patients presenting with behavior symptoms may have pathological features of AD. However, the relationship between* C9orf72* and the risk of AD remains controversial. Kohli et al. and Beck et al. demonstrated that* C9orf72* repeat expansions were risk factors for AD in the same year based on studies on large samples [13, 14]. However, Renton et al., Majounie et al., and some researchers stated that there were no associations between the gene mutation and the disease [5, 15].

*C9orf72* repeat expansions vary strongly between different geographic regions. In countries such as Italy, United States, and Germany, the prevalence can be as high as 47% for familial ALS and 21% for sporadic ALS [2, 5, 16]. However, in some Asian countries such as China and Korea, the mutation cannot be found in ALS patients [17]. Although* C9orf72* repeat expansions were considered pathological mutations of European ALS patients recently, the mutations were rare in Asians.

For better understanding the widening disease spectrum of* C9orf72* repeat copies, we performed a meta-analysis to clarify the association between* C9orf72* variations and AD or ALS.

## 2. Materials and Methods

### 2.1. Search Strategy

EMBASE, PubMed, and Cochrane databases were searched for the comprehensive literature (the search was conducted on May 20, 2015). We used search terms as follows: (“AD,” “Alzheime^*∗*^/$,” or “Alzheimer's disease”)/(“ALS,” “amyotrophic lateral sclerosis,” “motor neuron disease”) and (“*C9orf72*”). The languages of the articles were limited to Chinese and English. The strategies were made by two researchers (L. Shu and QY Sun). If there were any disputes, we consulted another researcher (JF Guo).

### 2.2. Selection of Studies

Studies were selected when they met the following criteria: (1) original articles (when there were secondary articles, we added the useful original articles from them); (2) association studies between* C9orf72* and AD or ALS; (3) sufficient data to calculate odds ratio (OR) and 95% confidence interval (95% CI); (4) study types being observational studies such as case-control studies and cohort studies; and (5) languages being limited to Chinese and English.

Studies were excluded when the following existed: (1) replicated data (if there were overlapped samples, we chose the largest samples); (2) incomplete data (no control group); (3) study types being reviews, case series, letters, editorials, and so forth; and (4) not human subjects (animal experiments, cell experiments, etc.). The PRISMA checklist is shown in Supplementary Table 1 (see Supplementary Table 1 in Supplementary Material available online at http://dx.doi.org/10.1155/2016/5731734).

### 2.3. Quality Assessment

The Newcastle-Ottawa Scale (NOS) was used to assess the quality of the selected original articles. The scale included three aspects: (1) subjects selection (0–4 points); (2) comparability between groups (0–2 points); and (3) exposure (0–3 points). The total points range from 0 to 9 [18]. The studies scored higher than 5 were considered of “high quality.” The assessments were conducted by two reviewers (L. Shu and QY Sun). The third author was consulted when agreements cannot be reached.

### 2.4. Data Extraction

Basic data were extracted for the statistical analysis including first author, publication year, ethnicity, country, age at onset, ages of cases and controls, genotyping methods, number of cases and controls, number of cases and controls who carried* C9orf72* repeat expansions or intermediate repeat copies, diagnostic criteria, and NOS scores. Data were selected by two researchers (L. Shu and QY Sun). The third author was involved to solve the disagreements.

### 2.5. Statistical Analysis

The correlation between* C9orf72 *and neurodegenerative diseases was analyzed by pooled OR with 95% CI. The heterogeneity among the included studies was estimated by *Q*-test or *I* ^2^ statistic. If the *Q*-test showed *p* value ≤ 0.1, the heterogeneity was considered significant. We chose fixed-effects model for statistical analysis with low heterogeneity while we chose random-effects model with moderate to high heterogeneity. *Z* test was conducted to measure the association between* C9orf72 *and neurodegenerative diseases. *p* value < 0.05 indicated statistically significant difference. Funnel plot was visually inspected to assess the possible publication bias [19]. Sensitivity analysis was conducted by sequentially removing one publication to evaluate the influence of single publication on the whole results. RevMan 5.2 software was used for all the statistical analyses and graphics.

## 3. Results

### 3.1. Association between* C9orf72* and AD

#### 3.1.1. Eligible Studies

After searching of PubMed, EMBASE, and Cochrane databases, 299 articles were identified. 89 duplicated datasets were removed and a total of 210 articles were reviewed by title and abstract. 190 articles did not meet the inclusion criteria and only 20 of them were reviewed for full-test assessment. Ten articles were excluded: 5 conference abstracts, 1 not peripheral blood test, and 4 without controls. Thus, there were 10 articles left for the final statistical analysis [13–16, 20–26] (the detailed flowchart was shown in Supplementary Figure 1).

#### 3.1.2. Characteristics of Studies

The detailed characteristics of the included studies are listed in Supplementary Table 2. From the table, there were 10 studies which had complete data about* C9orf72* expansions in both case and control groups and 3 studies about* C9orf72* intermediate repeat copies. The publication years of the included studies ranged from 2012 to 2014. The diagnostic criteria of AD were according to NINCDS-ADRDA criteria (Supplementary Table 2).

#### 3.1.3. Cumulative Analysis

**Figure 1:**
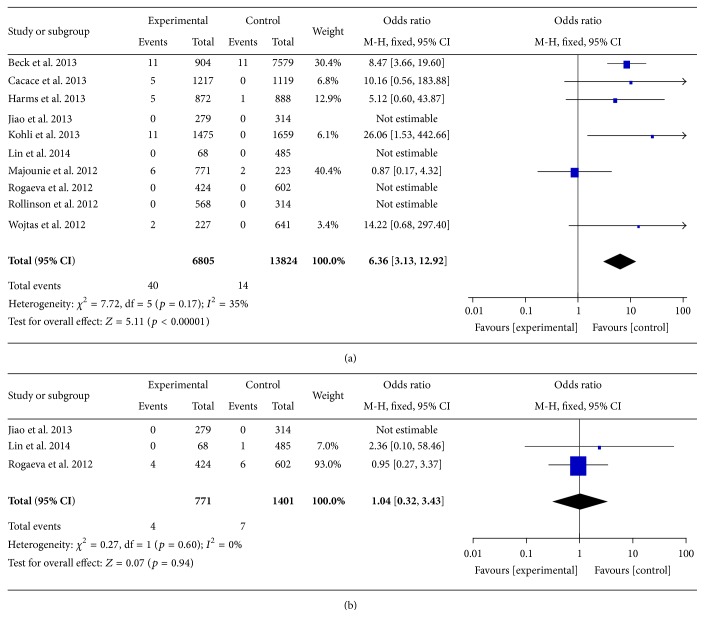
(a) Forest plot of the association between* C9orf72* repeat expansions and AD. (b) Forest plot of the association between* C9orf72* intermediate repeat copies and AD.

The results of the meta-analysis were present in Figures 1(a) and 1(b). There was no significant heterogeneity; thus a fixed-effects model was chosen for the meta-analysis. The results indicated that* C9orf72* repeat expansions were related to the risk of AD (OR = 6.36, 95% CI = 3.13–12.92, and *p* < 0.00001, Figure 1(a)) while* C9orf72* intermediate repeat copies were not correlated with the risk of AD (OR = 1.04, 95% CI = 0.32–3.43, and *p* = 0.94, Figure 1(b)).

### 3.2. Association between* C9orf72* and ALS

#### 3.2.1. Eligible Studies

We conducted a comprehensive search on PubMed, EMBASE, and Cochrane databases. After the initial search, we got 1224 articles. 413 repeated datasets and 738 articles were excluded by abstracts and titles. 73 articles were left for full-test review. Articles were further excluded for the following reasons: incomplete date (6 studies), no controls (16 studies), other diseases (7 studies), and so forth. Finally, 24 articles were left for the cumulative analysis [5, 14, 16, 17, 20, 26–45] (details were shown in Supplementary Figure 2).

#### 3.2.2. Characteristics of Studies

The details of the characteristics of the selected studies were present in Supplementary Table 3. There were 25 original articles included. Among them, 19 studies were conducted on Caucasians and 5 studies were performed on Asians. NOS scores of all the included studies are high except for three studies [5, 10, 33]. The publication years ranged from 2011 to 2014. The diagnostic criteria were according to El Escorial criteria or El Escorial revised criteria.

#### 3.2.3. Cumulative Analysis

After excluding three studies with low NOS scores, we conducted the cumulative analyses with 22 articles. Of these 22 articles, 18 articles containing complete data about familial ALS and 19 articles containing full data about sporadic ALS were included for meta-analysis separately. Subgroup analyses including 17 articles about Caucasians and 4 articles about Asians were conducted.

**Figure 2:**
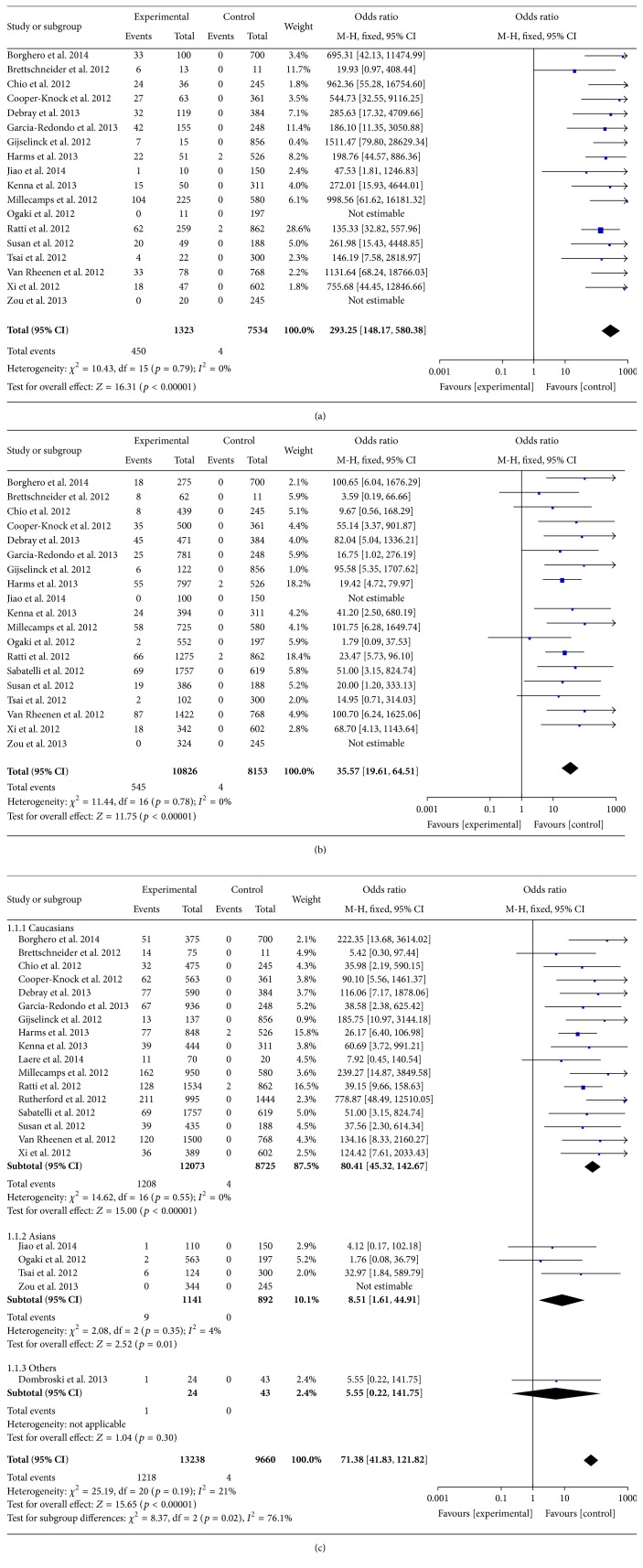
(a) Forest plot of the association between* C9orf72* repeat expansions and familial ALS. (b) Forest plot of the association between* C9orf72* repeat expansions and sporadic ALS. (c) Forest plot of the association between* C9orf72* repeat expansions and ALS (subgroup analysis).

The cumulative analyses suggested that* C9orf72 *repeat expansions were significantly correlated with the risk of ALS (OR = 71.38, 95% CI = 41.83–121.82, and *p* < 0.00001).* C9orf72 *repeat expansions were related to the risk of both familial and sporadic ALS (OR = 293.25, 95% CI = 148.17–580.38, and *p* < 0.00001; OR = 35.57, 95% CI = 19.61–64.51, and *p* < 0.00001). Subgroup analyses by ethnicity indicated that* C9orf72 *repeat expansions were risk factors for both Caucasian and Asian ALS patients (OR = 80.41, 95% CI = 45.32–142.67, and *p* < 0.00001; OR = 8.51, 95% CI = 1.61–44.91, and *p* = 0.01) (details were shown in Figure 2).

## 4. Sensitivity Analysis

We performed sensitivity analyses by omitting single articles to test the stability of the results. After sequentially omitting single studies about ALS and AD, the total results were similar.

## 5. Publication Bias

There were no asymmetries of the funnel plot below and no significant publication biases of the meta-analysis about* C9orf72* repeat expansions and AD or ALS (Supplementary Figure 3). We failed to do publication bias analysis on the association between* C9orf72* intermediate copies and the risk of AD because of the scarcities of the included articles.

## 6. Discussion

There were clinical, pathological, and hereditary overlap between FTD and AD; therefore some researchers regarded them as the diseases of the same disease spectrum [46]. After discovering the most common genetic cause of FTD-*C9orf72 *repeat expansions, many researchers began to focus on the association between* C9orf72 *and AD. However, the disputes have not been solved yet. The results of our meta-analyses demonstrated that* C9orf72 *repeat expansions were positively correlated with the risk of AD while* C9orf72 *intermediate repeat copies were not related to the risk of AD. However, the limited number of the original articles about the association between* C9orf72 *intermediate repeat copies and AD influenced the validity of the analysis. Additionally, the diagnostic standards of the patients in the original articles were based on the clinical diagnosis.

*C9orf72* has been discovered as the most common causative gene for ALS in white populations, which accounts for 40% of familial cases and 20% of sporadic cases in Finland [47]. While there were a large number of studies focusing on the identification of the gene in European countries, there were few studies reporting* C9orf72* mutation in Asian ALS patients. Our meta-analysis selected high-quality studies and demonstrated that* C9orf72* repeat expansions were related to the risk of ALS in Asians and Caucasians. We also proved that* C9orf72* repeat expansions were not only correlated with the risk of familial ALS but also related to the risk of sporadic ALS. However, there were evidences that anticipation played a role in* C9orf72* families. Gijselinck et al. demonstrated that there was a decreasing onset of age in younger generation in ALS families carrying longer G4C2 expansion. The original researches on the association between* C9orf72* repeat expansions and ALS can be invalid because of the genetic anticipation and the wide range of age of onset of* C9orf72* diseases [48]. Therefore, the different age of onset can cause bias in the meta-analysis of familial ALS. Additionally, there were few case-control studies about the relationship between* C9orf72* repeat expansions or intermediate repeat copies and ALS in other populations. Further large sample studies are essential to clarify the association.

Despite the limitations stated above, there were some other limitations. First, the* C9orf72* repeat expansions were analyzed by conventional PCR-based methods. When* C9orf72 *repeat copies were more than 60 copies, the accurate size cannot be gotten by conventional PCR-based methods. Other measures such as Southern blot were needed to detect the true repeat size [3]. Second, although our meta-analysis incorporated all the published case-control cohorts, some negative unpublished results were possibly neglected.

In conclusion,* C9orf72* repeat expansions were risk factors for AD while* C9orf72* intermediate repeat copies were not associated with the risk of AD.* C9orf72* repeat expansions were correlated with the risk of ALS. However, inevitable limitations existed in our meta-analysis such as the limited number of original articles and possible publication biases. Further assessments were needed with enough case-control samples.

## Supplementary Material

The flowchart of the selection of studies, the funnel plot and the detailed characteristics of the included studies are listed in the supplementary materials.
