# Supplementary material for: The Association between C9orf72 Repeats and Risk of Alzheimer's Disease and Amyotrophic Lateral Sclerosis: A Meta-Analysis
Source: Parkinsons Dis. 2016 Jun 8;2016:5731734. doi: 10.1155/2016/5731734 (PMC4916312; doi:10.1155/2016/5731734)
Supplement: Supplementary file 1 — The flowchart of the selection of studies, the funnel plot and the detailed characteristics of the included studies are listed in the supplementary materials. [file 5731734.f1.zip › Supplementary Fig. 3.pdf]

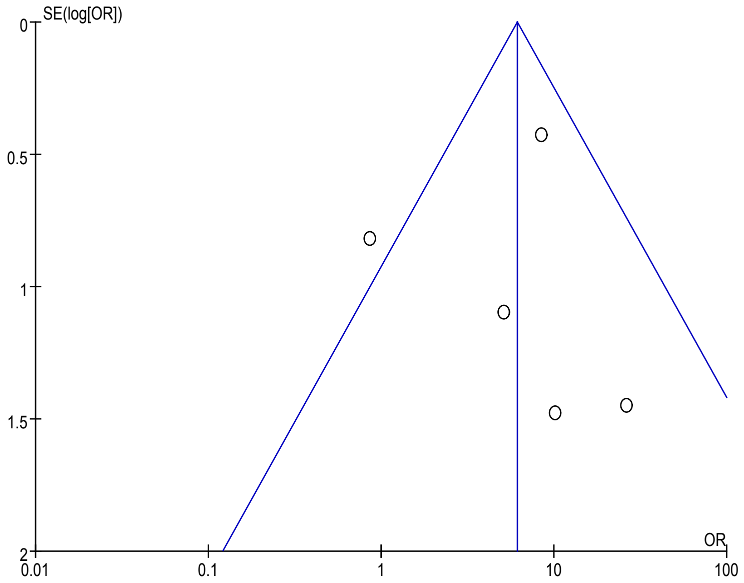

Fig. 3. (A) Funnel plot of the association between *C9orf72* repeat expansions and AD

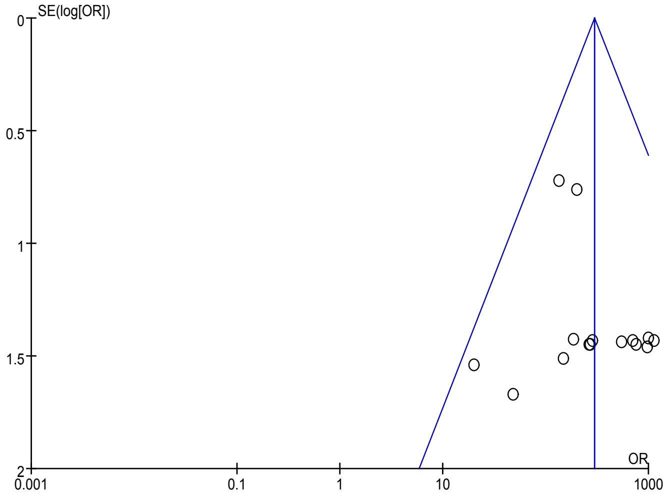

Fig. 3. (B) Funnel plot of the association between *C9orf72* repeat expansions and familial ALS

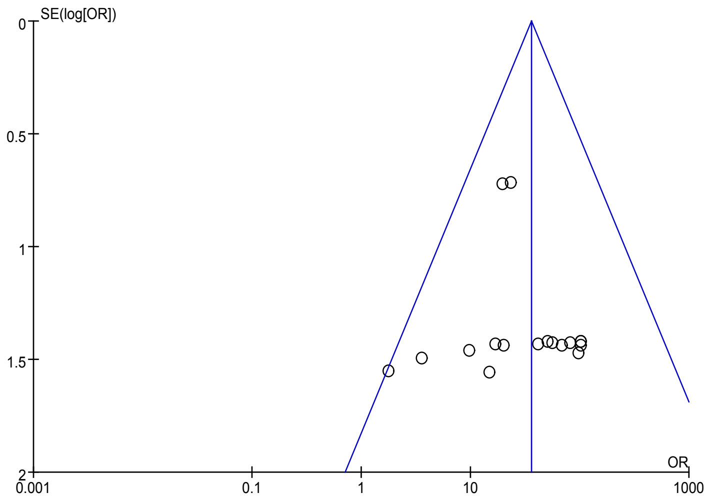

Fig .3. (C) Funnel plot of the association between *C9orf72* repeat expansions and sporadic ALS

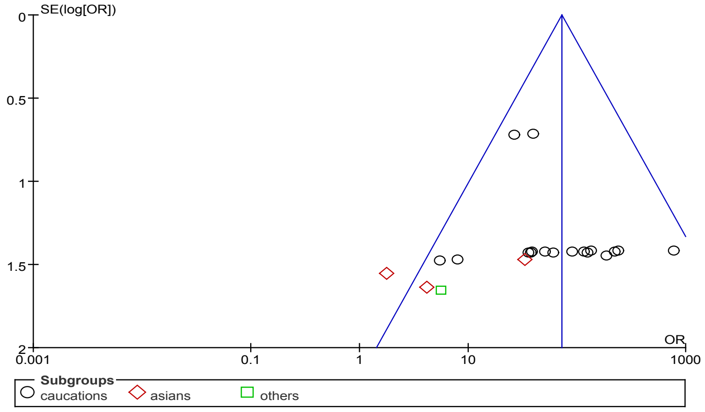

Fig. 3. (D) Funnel plot of the association between *C9orf72* repeat expansions and Caucasian and Asian ALS
