# Supplementary material for: The Association between C9orf72 Repeats and Risk of Alzheimer's Disease and Amyotrophic Lateral Sclerosis: A Meta-Analysis
Source: Parkinsons Dis. 2016 Jun 8;2016:5731734. doi: 10.1155/2016/5731734 (PMC4916312; doi:10.1155/2016/5731734)
Supplement: Supplementary file 1 — The flowchart of the selection of studies, the funnel plot and the detailed characteristics of the included studies are listed in the supplementary materials. [file 5731734.f1.zip › Table 2.pdf]

Table 1
Characteristics of the included studies for meta-analysis

Study	Ethnicity	Country	Age at onset (years)	Case age /Control age (years)	Genotyping method	Cases(familial /sporadic)	Controls(familial /sporadic)	Cases carry C9 repeat expansions(familial /sporadic)	Cases carry C9 intermediate repeats(familial /sporadic)	Controls carry C9 repeat expansions(familial /sporadic)	Controls carry C9 intermediate repeats(familial /sporadic)	Diagnostic criteria	NOS score
Majounie et al.(2012)			60-97		rpPCR	771(771/0)	223	6		2		NINCDS-ADRDA	5
Rogaeva et al.(2012)	Caucasian	European countries or North American countries	72.1±9.4		rpPCR	424(167/257)	602	0	4	0	6	NINCDS-ADRDA	7
Rollinson et al. (2012)				37-90/26-78	rpPCR	568	314	0	1	0		NINCDS-ADRDA	4
Wojtas et al.(2012)	Caucasian		59.8±7.0		rpPCR	227(128/99)	641	2		0		NINCDS-ADRDA	5
Beck et al.(2013)					rpPCR, Southern blot	904	7579	11		11			4
Cacace et al.(2013)	Caucasian	Flanders	74.5±8.9	/65.1±13.6	rpPCR	1217	1119	5(4/)		0		NINCDS-ADRDA	7
Harms et				75.8±	rpPCR,	872(872/0)	888	5(5/)		1(1/)		NINCDS-ADRDA	8

al.(2013)				8.9/71.41 ±6.7	southern blot									
Jiao et al.(2013)	Asian	China	70.1±10.0	72.4± 12.0/57.1 ±13.7	rpPCR	279(0/279)	314	0	0	0	0	NINCDS-ADRDA	6	
Kohli et al.(2013)	Caucasian /African	European countries/African American countries	70.84± 8.34/73.08 ±8.65	73.39± 7.71/69.54±6.83	rpPCR	1184/291	1039/620		11(11/0)		0(0/0)	NINCDS-ADRDA	8	/:different countries
Lin et al.(2014)	Asian	China	55.0±4.5	64.1± 4.5、50.6 ± 4.2/60.6 ±11.9	rpPCR	68(0/61+7)	485	0	0	0	1	NINCDS-ADRDA	4	
