# Supplementary material for: The Association between C9orf72 Repeats and Risk of Alzheimer's Disease and Amyotrophic Lateral Sclerosis: A Meta-Analysis
Source: Parkinsons Dis. 2016 Jun 8;2016:5731734. doi: 10.1155/2016/5731734 (PMC4916312; doi:10.1155/2016/5731734)
Supplement: Supplementary file 1 — The flowchart of the selection of studies, the funnel plot and the detailed characteristics of the included studies are listed in the supplementary materials. [file 5731734.f1.zip › Table 3.pdf]

Table 2
Characteristics of the included studies for meta-analysis

Study	Ethnicity	Country	Age at onset (years)	Case age/Control age (years)	Genotyping method	Cases(familial/sporadic)	Controls(familial/sporadic)	Cases carry C9 repeat expansions(familial/sporadic)	Cases carry C9 intermediate repeats(familial/sporadic)	Controls carry C9 repeat expansions(familial/sporadic)	Controls carry C9 intermediate repeats(familial/sporadic)	Diagnostic criteria	NOS score
Susan et al.(2012)	Caucasian	Ireland			rpPCR, Southern blot	435(49/386)	188	39(20/19)		0		El Escorial	7
Beck et al.(2013)					rpPCR, Southern blot	360	7579	29		11			4
Chio et al.(2012)	Caucasian	Italy			rpPCR	475(36/439)	245	32(24/8)		0		El Escorial	7
Debray et al.(2013)	Caucasian	Belgium			rpPCR	590(119/471)	384	77(32/45)		0		revised El Escorial	5
Dombroski et al.(2013)	others	Guam			rpPCR	24	43	1		0		El Escorial	7
Garcia-Redondo et al.(2013)	Caucasian	Spain			rpPCR	936(155/781)	248	67(42/25)	0	0	0	El Escorial	8
Gijselinck et al.(2012)	Caucasian	Flanders		59.9±11.6/65.3±14.8	rpPCR	137 (15/122)	856	13(7/6)		0		revised El Escorial	8
Harms et al.(2013)	Caucasian	US			rpPCR, Southern	51/797	526	77(22/55)		2		El Escorial	8

					blot						
Jiao et al.(2014)	Asian	China		41.1±11.5/39.7±8.9	rpPCR	110（10/100）	150	1(1/0)	0	El Escorial	6
Kenna et al.(2013)	Caucasian	Irish	61.7±12.0	/60.3±11.8	high throughput sequencing	444(50/394)	311	39(15/24)	0	revised El Escorial	7
Konno et al.(2013)	Asian	Japanese			rpPCR	168(58/110)	180	2(2/0)	0		3
Millecamps et al.(2012)	Caucasian	French			rpPCR	950（225/725）	580	162（104/58）	0	revised El Escorial	7
Ogaki et al.(2012)	Asian	Japanese	60.4±11.7	/60.6±10.3	rpPCR	563（11/552）	197	2（0/2）	0	revised El Escorial	7
Ratti et al.(2012)	Caucasian	Italy	57.1±12.7		rpPCR	1534(259/1275)	862	128（62/66）	2	revised El Escorial	8
Sabatelli et al.(2012)	Caucasian	Italy			rpPCR	/1757	619	69(0/69)	0	revised El Escorial	8
Tsai et al.(2012)	Asian	China			rpPCR	124(22/102)	300	6(4/2)	0	revised El Escorial	6
van Rheenen et al.(2012)	Caucasian	Netherlands		/63.1±11.8	rpPCR	1500(78/1422)	768	120(33/87)	0	revised El Escorial	8
Xi et al.(2012)	Caucasian	European countries or North American countries	57.6±12.3	/70.2±9.5	rpPCR	389(47/342)	602	36(18/18)	0	revised El Escorial	8

Borghero et al.(2014)	Caucasian	Italy		61.1±12.1/	rpPCR	375(100/275)	700	51(33/18)	0	revised El Escorial	8
Brettschneider et al.(2012)	Caucasian	America		55-69/60-78	rpPCR	75(13/62)	11	14(6/8)	0	revised El Escorial	7
Cooper-Knock et al.(2012)	Caucasian	Northern England			rpPCR	563(63/500)	361	62(27/35)	0	El Escorial	8
Laere et al.(2014)	Caucasian	Belgium	60.4±12.6		rpPCR	70	20	11	0	revised El Escorial	8
Rutherford et al.(2012)	Caucasian	America, British			rpPCR	995	1444	211	0	El Escorial	8
Zou et al.(2013)	Asian	China			rpPCR	344(20/324)	245	0	0	El Escorial	8
