# Supplementary figures and images for: The Association between C9orf72 Repeats and Risk of Alzheimer's Disease and Amyotrophic Lateral Sclerosis: A Meta-Analysis
Source: Parkinsons Dis. 2016 Jun 8;2016:5731734. doi: 10.1155/2016/5731734 (PMC4916312; doi:10.1155/2016/5731734)

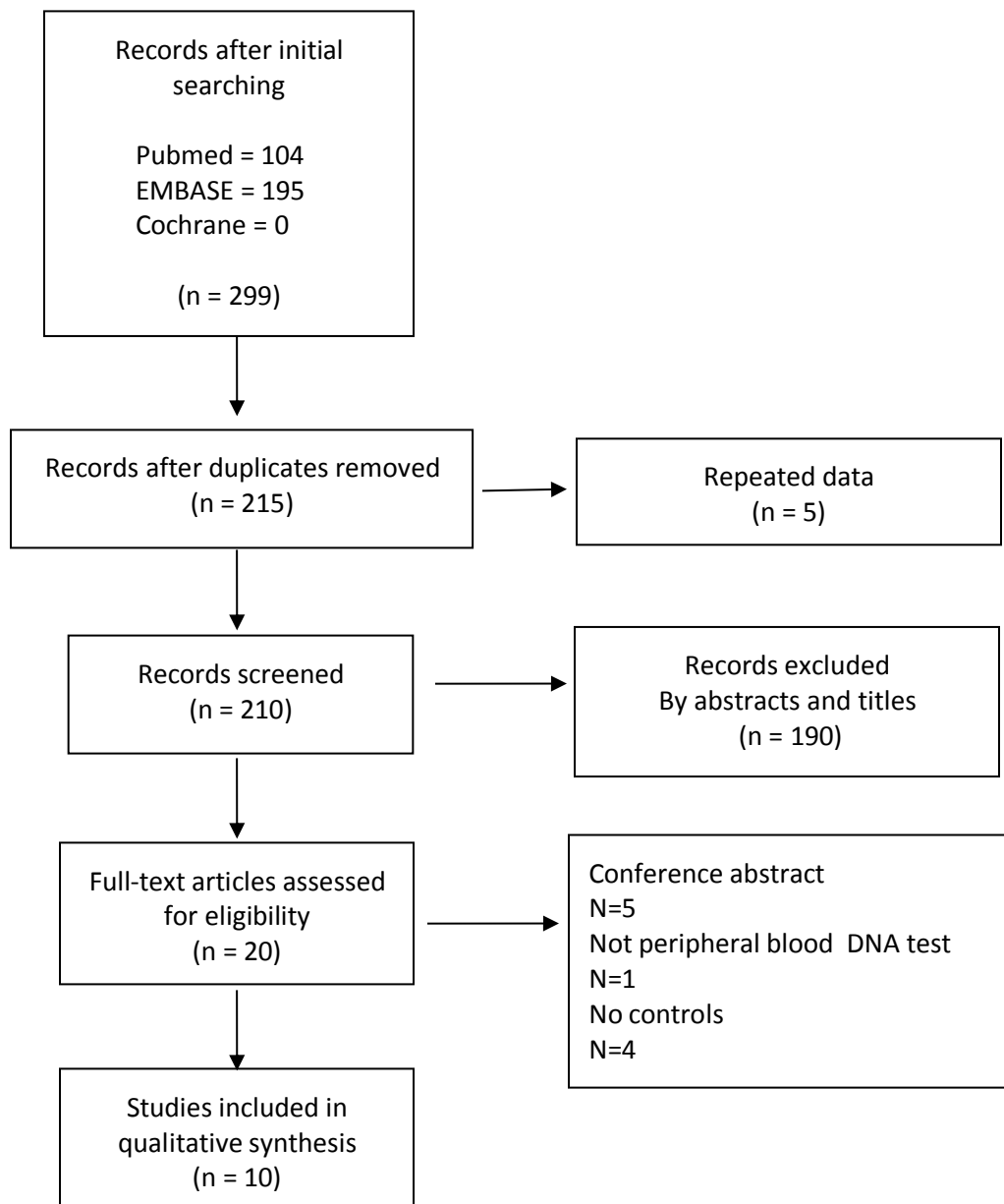

Fig. 1. Flowchart of the selection of studies (AD)

Supplement: Supplementary file 1 — The flowchart of the selection of studies, the funnel plot and the detailed characteristics of the included studies are listed in the supplementary materials. [file 5731734.f1.zip › Supplementary Fig. 1.pdf]

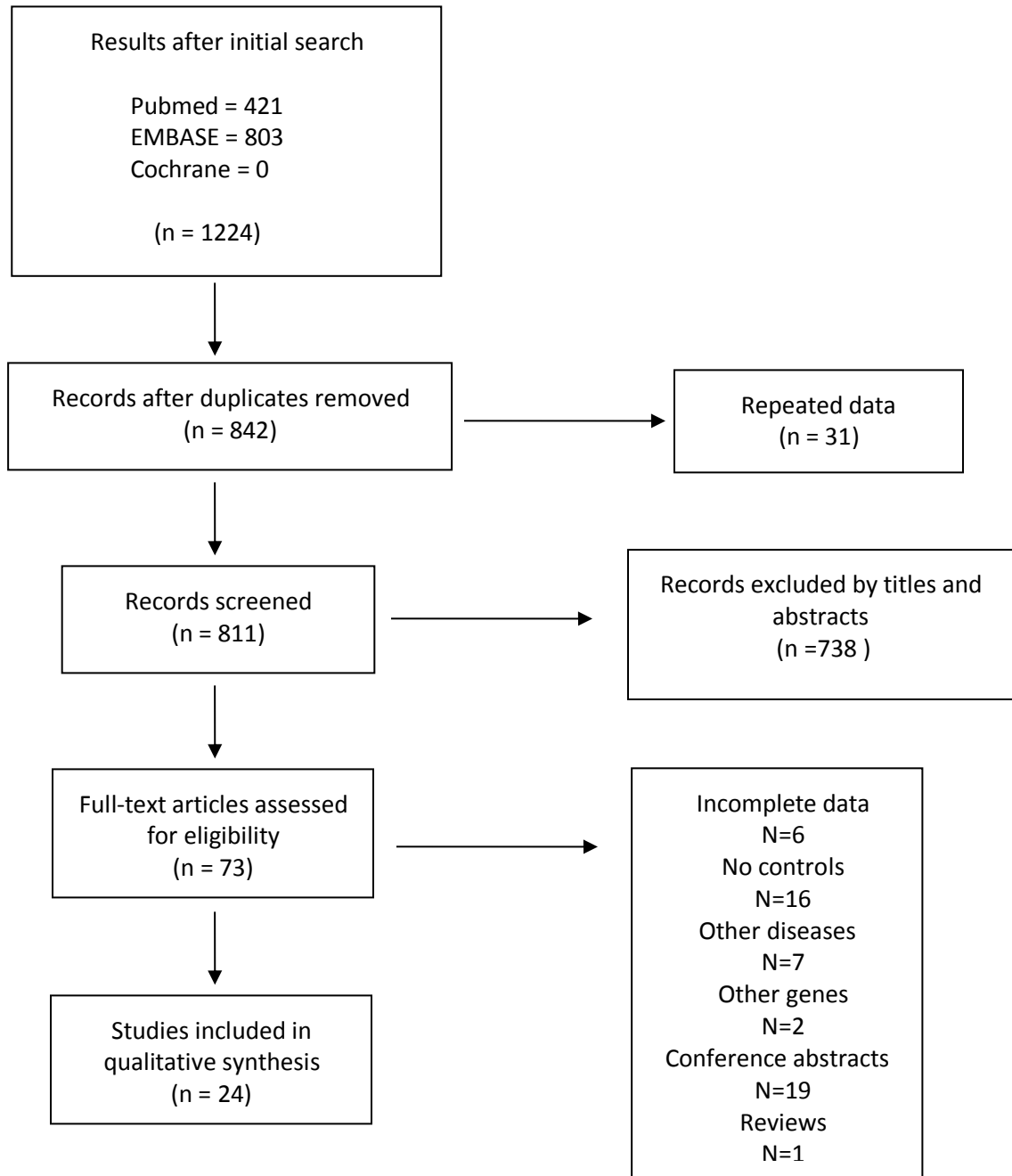

Fig. 2. Flowchart of the selection of studies (ALS)

Supplement: Supplementary file 1 — The flowchart of the selection of studies, the funnel plot and the detailed characteristics of the included studies are listed in the supplementary materials. [file 5731734.f1.zip › Supplementary Fig. 2.pdf]
